# Thomas Pullum & Co.

**Published:** 1876-04

**Authors:** 


THOMAS PULLUM & CO.—We hope our readers will not fail to notice in the present issue the card of Thomas Pullam & Co., wholesale druggists, Atlanta, Georgia.

We speak from personal knowledge, when we say this is among the best establishments in the whole country. The gentlemen who compose the firm have attained to high reputation in the community, being regarded as men of honor, integrity and fine business capacity.

